# Analysis of the Wear and Corrosion Resistance on Cu-Ni-Al Composites Reinforced with CeO_2_ Nanoparticles

**DOI:** 10.3390/ma18112438

**Published:** 2025-05-23

**Authors:** Carola Martínez, Bárbara Valverde, Aurora Del Valle-Rodríguez, Brennie Bustos-De La Fuente, Izabel Fernanda Machado, Francisco Briones

**Affiliations:** 1 Departamento de Ingeniería en Obras Civiles, Universidad de La Frontera, Temuco 4811230, Chile; b.bustos05@ufromail.cl; 2 Escuela de Ingeniería Mecánica, Facultad de Ingeniería, Pontificia Universidad Católica de Valparaíso, Quilpué 2430120, Chile; francisco.briones@pucv.cl; 3 Programa de Magister en Ciencias de la Ingeniería, Universidad de La Frontera, Temuco 4811230, Chile; aurora.delvalle@ufrontera.cl; 4 Departamento de Engenharia Mecatrônica e Sistemas Mecânicos, Escola Politecnica, Universidade de São Paulo, São Paulo 05508-030, Brazil; machadoi@usp.br

**Keywords:** Cu-Ni-Al alloys, CeO_2_ nanoparticles, scratch test, corrosion

## Abstract

This study evaluates the wear and corrosion resistance of the Cu-50Ni-5Al alloy reinforced with CeO_2_ nanoparticles for potential use as anodes in molten carbonate fuel cells (MCFCs). Cu–50Ni–5Al alloys were synthesized, with and without the incorporation of 1% CeO_2_ nanoparticles, by the mechanical alloying method and spark plasma sintering (SPS). The samples were evaluated using a single scratch test with a cone-spherical diamond indenter under progressive normal loading conditions. A non-contact 3D surface profiler characterized the scratched surfaces to support the analysis. Progressive loading tests indicated a reduction of up to 50% in COF with 1% NPs, with specific values drop-ping from 0.48 in the unreinforced alloy to 0.25 in the CeO_2_-doped composite at 15 N of applied load. Furthermore, the introduction of CeO_2_ decreased scratch depths by 25%, indicating enhanced wear resistance. The electrochemical behavior of the samples was evaluated by electrochemical impedance spectroscopy (EIS) in a molten carbonate medium under a H_2_/N_2_ atmosphere at 550 °C for 120 h. Subsequently, the corrosion products were characterized using X-ray diffraction (XRD), scanning electron microscopy coupled with energy dispersive spectroscopy (SEM-EDS), and X-ray photoelectron spectroscopy (XPS). The results demonstrated that the CeO_2_-reinforced alloy exhibits superior electro-chemical stability in molten carbonate environments (Li_2_CO_3_-K_2_CO_3_) under an H_2_/N_2_ atmosphere at 550 °C for 120 h. A marked reduction in polarization resistance and a pronounced re-passivation effect were observed, suggesting enhanced anodic protection. This effect is attributed to the formation of aluminum and copper oxides in both compositions, together with the appearance of NiO as the predominant phase in the materials reinforced with nanoparticles in a hydrogen-reducing atmosphere. The addition of CeO_2_ nanoparticles significantly improves wear resistance and corrosion performance. Recognizing this effect is vital for creating strategies to enhance the material’s durability in challenging environments like MCFC.

## 1. Introduction

Fuel cells (FCs) come in various types, differing in the electrolyte used, operating temperatures, and applications. The FCs are classified according to the operation temperature, the chemical nature of the electrolyte, and the fuel type [1]. The high-temperature technologies for generating electrical energy are molten carbonate fuel cells (MCFCs) and solid oxide fuel cells (SOFCs) [2] due to their high-power densities and efficiencies (100 MW) for stationary applications as power generators [3,4]. However, the MCFC operates at lower temperatures than SOFC, close to 650 °C, with a higher life span of 7000 h (SOFC 1000 h) [5], with flexibility of fuel, a low investment catalyst, and low cost [6]. This is why it is considered an attractive option to develop medium-scale stationary units that generate between 100 kW and 100 MW [7]. MCFC comprises an anode and a cathode separated by a molten carbonate electrolyte. The primary issues with MCFC are the stability and degradation of materials caused by high temperatures, which decrease cell efficiency [8].

Ni-Al anodes are widely used in MCFC applications due to their relatively low density, high melting point, excellent corrosion resistance to acids and alkalis, and good charge transfer conductivity [9]. Anode materials are typically Ni–Cr/Ni–Al alloys [10,11], and cathode materials are comprised of lithiated NiO [12]. The fuel cell operation results from a complex conjunction of physical, chemical, and electrochemical processes. The anode and cathode half reactions and overall electrochemical reaction are [13,14]:

Cathode: ½ O_2_ + CO_2_ + 2e^−^→ CO_3_^2−^

Anode: H_2_ + CO_3_^2−^ → H_2_O + CO_2_ + 2ē

Overall: H_2_ + ½ O_2_ + CO_2 (cathode)_ → H_2_O + CO_2 (anode)_ + electricity + heat

Due to the high temperature, they are susceptible to creep, which causes a decrease in sintering resistance and a reduction in electrode pore size, dramatically shortening the lifespan of fuel cells [9,15]. In this context, generating efficient and stable anodes by incorporating alloy additives requires understanding the underlying mechanisms to design other efficient and stable anodes. For this reason, incorporating other metals, such as copper, was investigated to enhance structural stability [16,17].

The incorporation of copper (Cu) into the Ni-Al alloy presents an opportunity to enhance thermal conductivity and mechanical strength over a wide range of temperatures, making it an ideal material for operating under extreme conditions of heat flow and compression [10,17]. Copper is less expensive and more stable under accidental current overload than nickel because of its positive potential. Furthermore, copper has higher electrical conductivity and greater resistance to carburization than nickel [16]. According to Klassert and Tikana [11], copper alloys are an important metallic material due to their metallurgical, physical, and chemical properties. For example, Cu-Ni alloys show high tensile strength and good corrosion resistance at high temperatures. Other authors, such as Martínez et al. [18], study Cu and Ni separately, as well as the Cu-Ni alloy, indicating that Ni exhibits the maximum resistance. However, the Cu-Ni alloy has intermediate values between Cu and pure Ni. It is essential to mention that the incorporation of metal can stabilize the alloy in a solid solution, which means that solvent metal atoms are randomly replaced by solute metal atoms in their positions in the lattice, creating metallic bonds [19,20].

Alternatively, the incorporation of nanoparticles in commercial alloys, such as Ni-5Al, has been studied to enhance the mechanical behavior of the anode, incorporating zirconia oxides (ZrO_2_) [21,22] and cerium oxides (CeO_2_) [23]. The addition of nanoparticles to the Ni-5Al alloy reduces creep deformation by enhancing microstructural stability, catalytic activity, and cell efficiency [18]. Some authors have reported that incorporating CeO_2_ nanoparticles enhances the catalyst performance at the anode for the H_2_ oxidation reaction [22,23]. Additionally, incorporating cerium oxide (CeO_2_) nanoparticles into Cu-Ni alloys has improved the catalyst performance in the hydrogen oxidation reaction [24]. Recently, studies have focused on the effects of hydrogen atmospheres on the durability and efficiency of fuel cell components. For example, research by Accardo et al. [22] demonstrated that exposure to hydrogen can lead to the formation of nickel hydrides on the anode surface, affecting its electrochemical properties. Furthermore, hydrogen embrittlement is a phenomenon where materials become brittle due to hydrogen absorption, posing a significant challenge to the structural integrity of fuel cell components [22]. However, further research is still required on the micro- and macrostructural behavior and corrosion resistance of these nanoparticle-reinforced alloys in hydrogen atmospheres.

On the other hand, there are few studies on the tribological behavior of the components used in a fuel cell (FC). Although MCFC anodes are not subject to continuous mechanical friction during operation, their surfaces may suffer localized damage during stack assembly or thermal cycling. Scratch resistance is an indicator of surface hardness and mechanical stability, which helps preserve the anode’s porosity and functionality. Recent studies have shown that enhancing wear resistance in functional surfaces, even without active sliding, contributes to improved durability and electrochemical performance [25,26]. Venkatesh et al. [27] evaluate friction wear damage in a single microcontact at different contact pressures on a proton-exchange membrane fuel cell. However, microcontacts are likely to occur during service in the fuel cell assembly, which can affect fuel cell performance. V. E. Pukha et al. [28] investigate CNC-coated titanium as a viable alternative to gold-coated stainless steel for bipolar plates in proton exchange membrane fuel cells, focusing on its corrosion, low interface contact resistance (ICR), and high wear resistance. However, it is expected that CeO_2_ nanoparticles, due to their lubricating properties [29], could decrease the coefficient of friction, thereby improving tribological performance by reducing wear and increasing the useful life of the anodes. The above is considering that the addition of CeO_2_ nanoparticles to copper-based alloys can simultaneously enhance their mechanical, tribological, and thermal stability properties. At the microstructural level, CeO_2_ acts as a grain refiner and a barrier to dislocation motion, contributing to increased hardness and improved high-temperature creep resistance [30]. From a tribological perspective, nanoscratch and wear studies have demonstrated that CeO_2_ facilitates the formation of stable and homogeneous tribochemical films, reducing friction and protecting the surface from progressive wear [31]. In addition, in spark plasma sintered composites such as CeO_2_-doped Cu/WS_2_, these nanoparticles help control interfacial microstructure and prevent thermal degradation during processing, leading to materials with high densification, low friction, and good mechanical strength [32].

Therefore, this work aims to analyze the influence of CeO_2_-NPs as a reinforcing material in Cu-Ni-Al alloys in the singular scratch test and the corrosion resistance in molten salt of carbonate in an H_2_/N_2_ atmosphere, in order to analyse their possible use as an anode in a molten carbonate fuel cell (MCFC).

## 2. Materials and Methods

### 2.1. Samples Manufacturing

Sample manufacturing was performed using mechanical alloying and mechanical mixing. Mechanical alloying was performed with pure powders: Cu (99.7% at., <63 mesh, Merck, Darmstadt, Germany), Ni (99% at., <230 mesh, Merck), and Al (<60 μm, 99.9%, Good Fellow, Peterlee, UK). The mechanical alloying was carried out with a powder ratio of Cu-50Ni-5Al (wt.%) in a planetary mill (Across International model PQ-N04, Bayswater, VIC, Australia) using stainless steel containers and balls (ball diameters: 10 and 6 mm). The containers were filled in a glove box under an argon atmosphere. The ball-to-powder ratio (BPR) was 10:1, and 2 wt.% stearic acid was used as a control agent. The angular velocity is 350 r.p.m., and a cycle of 30/15 min on/off is used to maintain a constant temperature over a 30 h milling time.

The 1% CeO_2_ nanoparticles (CeO_2_-NPs) were incorporated in Cu-50Ni-5Al in a mixed Y–type Astecma for 1 h. Subsequently, Cu-50Ni-5Al samples without and with CeO_2_-NPs were consolidated by Spark Plasma Sintering (SPS) using a Fuji Electronic Industrial Co. model DR SINTER SPS1050 (Tsurugashima, Japan), according to the procedure previously described by Martínez et al. [18]. The samples obtained have a 16 ± 0.8% porosity for Cu-50Ni-5Al and 1.0 ± 0.3% for Cu-50Ni-5Al + 1 wt.% CeO_2_-NPs; and the hardness values of 0% CeO_2_–NPs is 205 ± 21 HV_0.3_ and 1% CeO_2_–NPs is 340 ± 37 HV_0.3_ [18].

A Mecatome–Presi machine mechanized Cu-Ni-Al and Cu-Ni-Al + 1% CeO_2_-NPs samples. Then, a Dremel was used to make a hole in the top of the samples for electrochemical analysis.

### 2.2. Singular Scratch Test

The samples were metallographically prepared by reducing the grit size of the abrasive from #800, #1000, to #1200 SiC sandpaper. Subsequently, automatic polishing was performed using a Struers polisher, model Labopol-60 (Champigny-sur-Marne, France), with cloths for the different suspensions of diamond particles (diamond paste) of 6, 3, and 1 µm. To obtain the final surface, the sample was polished on colloidal silica (0.04 µm). Finally, to eliminate particles on the sample surfaces, they were left in alcohol under ultrasound and dried in air.

Scratch tests at this scale were used with a Bruker UMT-2 Tribometer (Billerica, MA, USA). The scratching procedures were performed using a Rockwell-C spherical diamond indenter (Milwaukee, WI, USA) with a radius of 200 µm and a 120° spherical radius—the test protocol employed a progressive loading Scratch Test. The normal load was increased linearly from 5 to 35 N during the test. The coefficient of friction is the ratio of the tangential force (F_t_) to the normal force (F_n_). The curve plotted with different loads is an average of three scratch measurements. The profiles of wear tracks were analysed using the 3D interferometry technique with the Taylor-Hobson model CCI MP equipment (Leicester, UK). Each scratch was inspected at three positions between the starting and end points to measure the pile-up area (Force normal: 7 N, 23 N and 33 N), scratch area, and scratch depth. The CCI-MP performs area measurements on the mean profile of the scratch section appearing in the field of view and provides the average values. The data obtained were analyzed using the software Talymap Gold v6.2.661.

### 2.3. Electrochemical Measurements

The electrochemical behavior of the Cu-50Ni-5Al samples was studied using open circuit potential and electrochemical impedance spectroscopy (EIS) measurements in the molten Li_2_CO_3_-K_2_CO_3_ (62:38 mol. %) as electrolyte at 550 ± 5 °C under a controlled media were performed using a constant flow of H_2_/N_2_ in a 40/10 mL·min^−1^ ratio. The electric contact for the working electrode, the Cu-50Ni-5Al samples, was performed using conductive silver printing ink (resistivity 5–6 µΩ cm) around the sample and copper wire of 25 cm in length. In addition, a Pt wire was used as the counter electrode, measuring 25 cm in length. An Ag wire, also 25 cm in length, was placed inside a quartz glass tube with a porous plug at the tip, serving as the reference electrode. The electrochemical measurements were performed using a Potentiostat from Solartron Analytical (Farnborough, UK).

**Figure 1:**
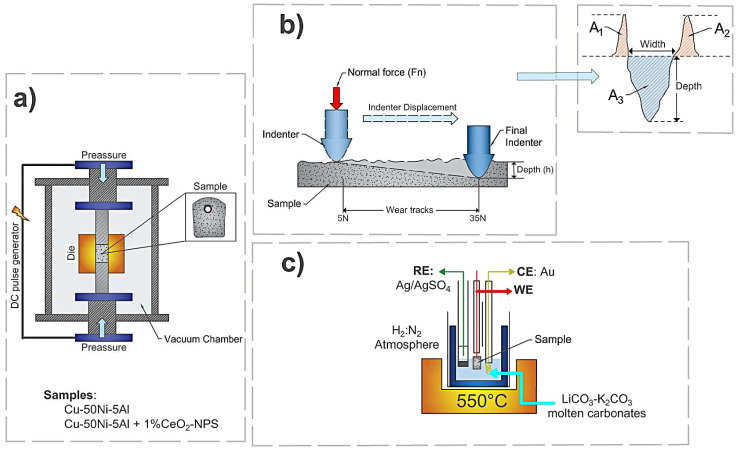
Schematic representation of the methodology: (**a**) SPS system (consolidation), (**b**) single scratch test, and (**c**) set-up of half-cell to Li_2_CO_3_-K_2_CO_3_ at 550 °C under an H_2_/N_2_ atmosphere.

Figure 1 provides a summary diagram illustrating the methodology employed in this study.

### 2.4. Morphological and Microstructural Analysis

Morphological characterization was performed using scanning electron microscopy (SEM) with a Hitachi SU3500 microscope (Tokyo, Japan) equipped with an EDX analyser from Bruker XFlash, 15 kV accelerating voltage, and 155.000 nA current. The samples were characterized by X-ray diffraction (XRD) to identify phases and assess phase purity. X-ray powder diffraction patterns were obtained using a powder diffractometer Bruker D2 Phase equipped with a Cu-K_α_ radiation source. The diffraction patterns were recorded with a 2θ range of 20° to 100°, a step size of 0.02°, and a counting time of 1 s per step. Diffraction patterns were analyzed using MACH! 3 Software version 3.15 build 258 to identify the phases present. X-ray photoelectron spectroscopy (XPS) was also performed to study the surface composition in more detail, utilizing an analysis chamber, Phoibos 150 (SPECS Group, Berlin, Germany), equipped with a 1D-DLD detector and a monochromatic X-ray source: Focus 500 with an XR50 M, featuring an Ag/Al Anode and X-ray source power of 400 W. The XPS data were analyzed using Casa XPS Version 2.3.22 software, calibrating the energy scale of the spectra with the binding energy of the C1s signal at 285.4 eV, which allowed us to remove the Shirley-type background. A mixed Gauss–Lorentz shape was used for the different components.

## 3. Results

### 3.1. Scratch Test with Progressive Normal Load

**Figure 2:**
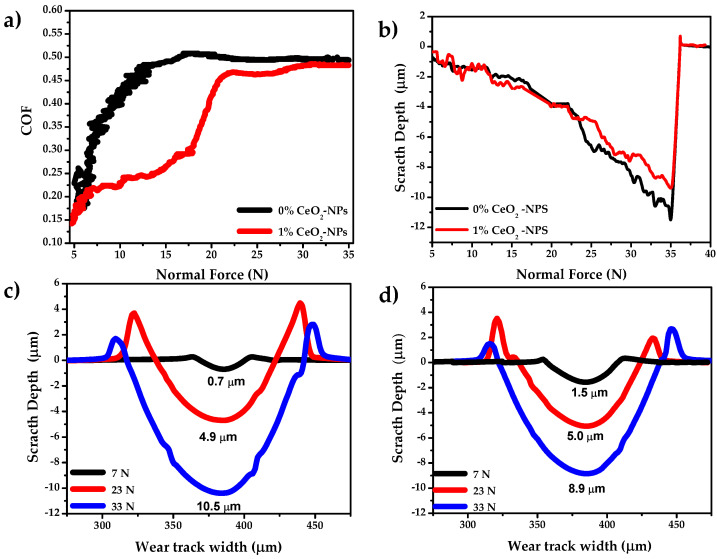
(**a**) COF vs. normal Force, (**b**) normal force vs. scratch depth, and the wear tracks profile (**c**) 0% CeO_2_–NPs, and (**d**) 1% CeO_2_-NPs.

Figure 2 presents the scratch test results for Cu-50Ni-5Al + 0% CeO_2_-NPs (0% CeO_2_-NPs) and Cu-50Ni-5Al + 1% CeO_2_-NPs (1% CeO_2_-NPs) samples. Figure 2a shows the coefficient of friction (COF) as a function of normal force (Fn). The average coefficient of friction (COF) values obtained for the three normal loads evaluated show consistent differences between the samples with and without the addition of CeO_2_-NPs. In the unreinforced alloy, values of 0.24 ± 0.06 (7 N), 0.49 ± 0.02 (23 N), and 0.49 ± 0.02 (33 N) were recorded, while in the samples with 1% CeO_2_, values of 0.22 ± 0.04 (7 N), 0.46 ± 0.03 (23 N), and 0.48 ± 0.01 (33 N) were obtained. The observed differences remain consistent at all load levels. It is important to highlight that the dispersion levels are within the ranges typically reported for metallic materials evaluated by scratch tests, as pointed out in previous studies [33].

The 0% CeO_2_-NPs sample exhibits more significant COF oscillations up to approximately 13 N. Then, the friction curve increases and stabilizes at approximately 21 N and remains without significant changes in the slope as the force reaches the maximum load of 35 N.

For the 1% CeO_2_-NPs, small oscillations are observed in the coefficient of friction (COF) curve up to approximately 7 N. After this point, there is a significant change in the slope of the curve that extends to 17 N, followed by another increase in the slope up to 22 N. This behavior may be associated with a transition region characterized by plastic deformations occurring below, besides, and in front of the tip [34]. Around 23 N, the COF curve shows no significant changes in slope, resulting in the same COF values for the 0% CeO_2_-NPs sample. This phenomenon is attributed to the material removal, which alters the tangential force proportionately to the normal force. It is important to note that between 7 N and 23 N, there are significant differences in the COF between the two samples. Specifically, the sample containing 1% CeO_2_-NPs shows a remarkable reduction in COF of more than 50%, reaching a value of 0.25 ± 0.01 at 15 N. In contrast, the 0% CeO_2_-NPs sample has a COF of 0.48 ± 0.02. This decrease in COF can be attributed to the presence of load-bearing nanoparticles, which reduce the actual contact area and the interaction of asperities between the contacting surfaces. This interaction varies due to differences in hardness between the samples and the presence of pores during the sliding of the indenter on the surface where the indenter penetrates deeper, increasing the friction force [35] (0% CeO_2_-NPs: 16 ± 0.8% de porosity; 1% CeO_2_-NPs: 1.0 ± 0.3% porosity [18]).

Figure 2b shows the scratch depth curves as a function of normal load. Starting at 23 N, the results indicate a separation between the two curves, reaching an average difference between depths of 25%. Due to these transitions, three normal loads, specifically 7, 23, and 33 N, were selected for further analysis.

Figure 2c and 2d shows the profile of the wear track and the pile-up of both samples, indicating that when the indenter (hard particle) scratches the alloy containing 1% CeO_2_-NPs, it produces a shallower scratch. These results indicate that the sample with 0% CeO_2_-NPs will experience greater mass loss than 1% CeO_2_-NPs. It is important to note that higher hardness is directly correlated with shallower scratch depths, which aligns with the general principle that harder materials exhibit better resistance to wear and abrasion [36] (0% CeO_2_–NPs: 205 ± 21 HV_0.3_ and 1% CeO_2_–NPs: 340 ± 37 HV_0.3_ [18]). This observation is consistent with widely reported findings in the literature showing that increased surface hardness reduces plastic deformation and the material removal rate during contact, which ultimately improves wear resistance. Numerous metallic and composite systems studies support this correlation [37,38,39]. Furthermore, applying higher normal loads is expected to increase the scratch depth. On a microscale, this interaction varies within a specific range throughout the test period due to hardness differences between samples and the presence of pores during the sliding of the indenter on the surface [35].

Another important aspect is the accumulation of material along the edges of the scratch track, which is evident in all samples under different applied loads. This accumulation of displaced material indicates significant plastic deformation during the tip-sliding process. The pile-up formation suggests the material exhibits a relatively low strain-hardening capacity, allowing localized plastic flow rather than extensive fracture or substantial material removal. These effects were more pronounced at a load of 23 N in both cases, which is attributed to the fact that the magnitude of pile-up is influenced by both the applied load and the material hardness [40]. The more pronounced pile-up formation in the 0% CeO_2_–NPs sample could be attributed to its higher porosity, which reduces the local hardness and favors material displacement rather than fragmentation. In contrast, the lower stacking height in the 1% CeO_2_–NPs sample is probably due to the effect of the nanoparticles, which contributes to higher hardness, resistance to localized deformation, and lower presence of pores during tip sliding.

Table 1 presents the wear profiles’ main area and dimension parameters for the three selected loads. The first ratio, A_1_+A_2_, is associated with the pile-up, and the A_3_ value is associated with the material that originally occupied the tracks. Attributing its destination to the formation of piles and wedges or effective removal by micro-cutting is unnecessary. These area ratios are justified by considering practical situations in which the portion of material that has moved out of the tracks (pile and wedge) can be removed with increases in normal force. Therefore, the ratio of these areas can suggest predominant micro-mechanisms or transitional micro-mechanisms [41], where it is observed that relationships close to 0 indicate micro-cutting as the predominant micro-mechanism (values of 33 N), and values very close to 1 indicate micro-ploughing as dominant at low loads (7 N). Between these values, there would be a transition of micro-mechanisms. Additionally, the main differences between the results in A_1_+A_2_ in the profiles at 23 N could be associated with the fact that the undetected chips cause an increase in the measurements of the stacking areas, where Franco et al. [42] address these false accumulations, which is a delicate point in area measurement.

**Figure 3:**
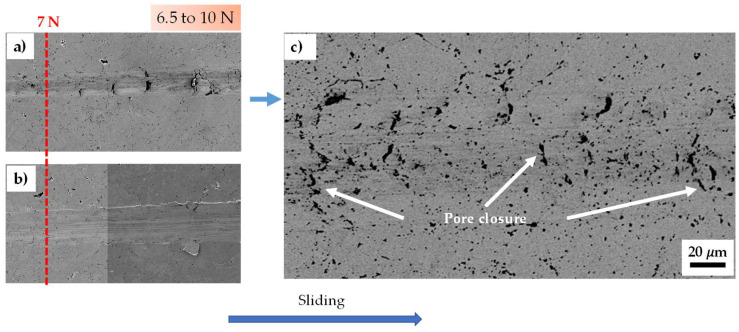
Wear path of the (**a**) 0% CeO_2_-NPs, (**b**) 1% CeO_2_-NPs, and (**c**) zoom in the wear track 0% CeO_2_-NPs. Applied normal force: between 6.5 and 10 N.

Figure 3 shows SEM images of both samples between 6.5 to 10 N of applied load. Generally, during the indenter passage, most of the material appears to sink to the bottom of the tracks, and a small amount shifts to the edges, forming the pile-up. This micro-mechanism is well known in the literature as micro-ploughing [43]. Figure 3c shows the wear track of the 0% CeO_2_-NPs sample at higher magnification. The figure shows the presence of pores, in which the loading conditions may tend to cause the closure of surface pores during tip sliding, which influences how the material deforms during the test [44]. The tendency towards pore closure may explain the differences in the geometric parameters of the wear profile between both samples at 7 N (see Table 1), causing the increase in contact pressure that promoted high plastic deformation around the surface pores. This localized deformation induced the surface pores to collapse instead of moving towards the edges and bottom of the tracks [44,45].

**Figure 4:**
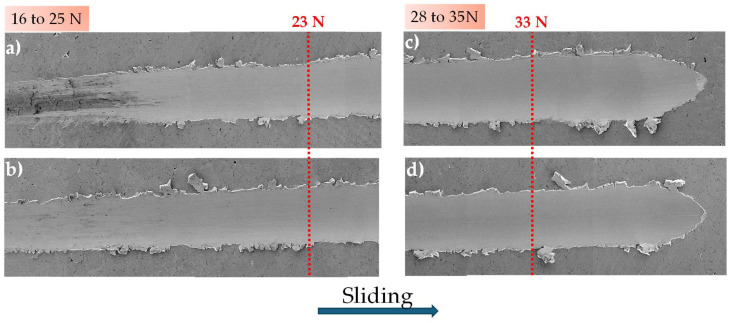
Wear path of the (**a**,**c**) 0% CeO_2_-NPs, (**b**,**d**) 1% CeO_2_-NPs. Applied normal force: between 16 to 25 N and 28 to 35 N.

Figure 4 shows the wear path for distinct applied normal force. At 16 to 25 N normal force (Figure 4a,b), the track becomes wider and exhibits more intense plastic deformation within the track. A micro-mechanism transition can be seen from micro-ploughing to micro-cutting, where the accumulation of material on the edges generates the formation of chips as the normal load increases. This micro-mechanism of transition from mild to severe abrasion often occurs similarly in both materials. The transition is attributed to the fact that in ductile materials, it is common to find chips that do not detach from the surface and remain near the edge of the track [42], as seen in both SEM images, which supports the findings presented in Table 1.

At loads of 28 and 35 N, a more pronounced plastic deformation is observed within the tracks (see Figure 4c,d). In addition, it is seen that there are regions with more intense interaction between the indenter and the surface, typical of micro-cutting: chip formation, chip detachment, and chips next to the track. Both tracks show significant width variations and regions with poorly defined edges. This could explain the differences in parameter A_3_ between both samples, which show significant differences; the 1% CeO_2_-NPs sample has a 20% smaller area than the 0% CeO_2_-NPs sample. The use of this parameter is justified considering practical situations in which the portion of the material that has moved outside the track is easily removed at those load levels (33 N). As a result, the 0% CeO_2_-NPs sample shows more material displaced outside the track and greater penetration depths (Figure 2). These values indicate the advantages of using nanoparticles at high normal force.

### 3.2. Electrochemical Measurements

**Figure 5:**
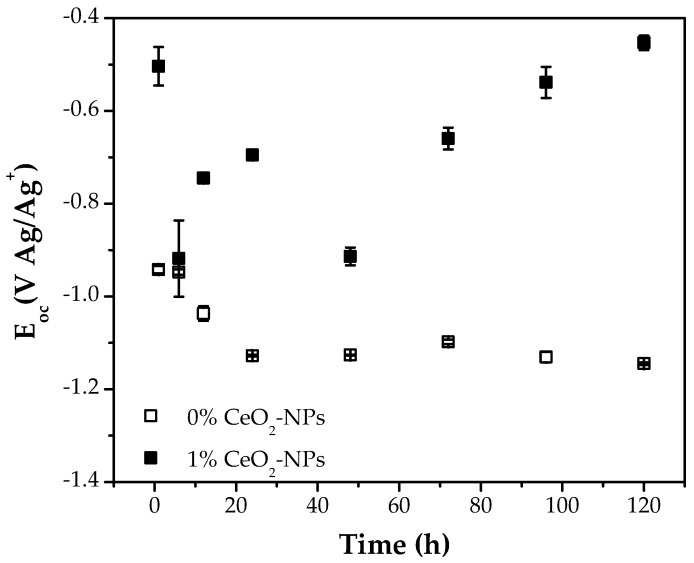
Open circuit potential variation of (□) 0% CeO_2_-NPs and (■) 1% CeO_2_-NPs during exposure to Li_2_CO_3_-K_2_CO_3_ at 550 °C under an H_2_ atmosphere.

Figure 5 shows the open circuit potential (E_OC_) of the effect of 0% CeO_2_-NPs and 1% CeO_2_-NPs after exposure to Li_2_CO_3_-K_2_CO_3_ at 550 °C in H_2_/N_2_ atmosphere. After a shorter exposure time, the E_OC_ shifted to more negative values upon incorporating CeO_2_-NPs, suggesting an activation of corrosion phenomena. However, for longer exposure times, the E_OC_ reached potential values above the sample without CeO_2_-NPs of approximately −0.43 V vs Ag/Ag+, which can be attributed to a stable oxide layer formed on the metal surface [46]. The 0% CeO_2_-NPs sample exhibits a decrease in the E_OC_ at shorter exposure times; however, after 20 h, relatively constant values are observed. This behavior can be attributed to Al, forming a protective alumina layer on the surface, preventing continued reaction at higher temperatures [47].

**Figure 6:**
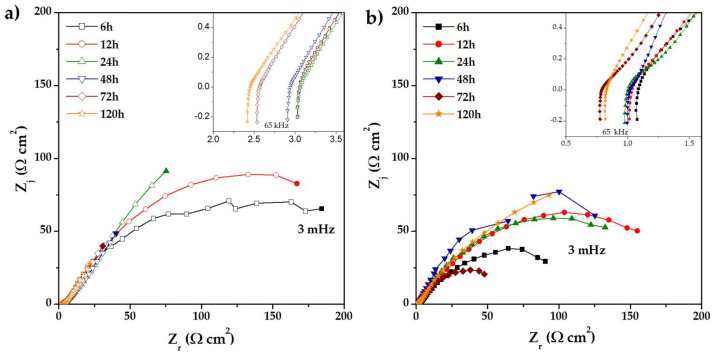
Nyquist diagram of (**a**) 0% CeO_2_-NPs, and (**b**) 1% CeO_2_ NPs during exposure to Li_2_CO_3_-K_2_CO_3_ at 550 °C under an H_2_/N_2_ atmosphere.

Figure 6 shows the Nyquist diagrams of 0% CeO_2_-NPs and 1% CeO_2_-NPs samples after 120 h of exposure to Li_2_CO_3_-K_2_CO_3_ in a H_2_ atmosphere at E = E_OC_ and 550 °C. The impedance analysis reveals a capacitive behavior of the alloys, independent of CeO_2_-NPs, indicating a higher resistance to polarization as a function of increased exposure time for both samples, attributed to the formation of protective oxides [18]. Concerning the effect of temperature on the reaction rate, the anodic reaction has positive activation energies [48], indicating that the oxidation of H_2_ is facilitated at higher temperatures. The heat of the reaction for the gaseous dissolution of H_2_ and CO_2_ in carbonates also has positive values [49]. Therefore, a higher temperature increases the rate of gas dissolution.

H_2_ + CO_3_^−2^ → CO_2_ + H_2_O + 2ē (1)

½ O_2_ + CO_2_ + 2ē → CO_3_^−2^ (2)

The incorporation of 1% CeO_2_-NPs decreases the resistance to polarization, which is observed in a less capacitive arc at all exposure times; this may be associated with the protective properties of the oxide layer formed on the surface, such as its poor adhesion and heterogeneity [50]. It should be noted that at 120 h, the 1% CeO_2_-NPs sample presents better behavior than the 0% CeO_2_-NPs sample. This can be attributed to the difference in the corrosion products formed between the two systems, which reduces electrochemical activity and cell performance.

As can be observed from E = E_OC_, the impedance responses reveal two-time constants in the high- and low-frequency ranges (HF and LF), which can be associated with the cathodic current. This current involves the capacitance of the electric double layer (C_dl_) and the formation of an oxide layer due to the dissolution of the alloy, which is related to the surface’s heterogeneity [51].

**Figure 7:**
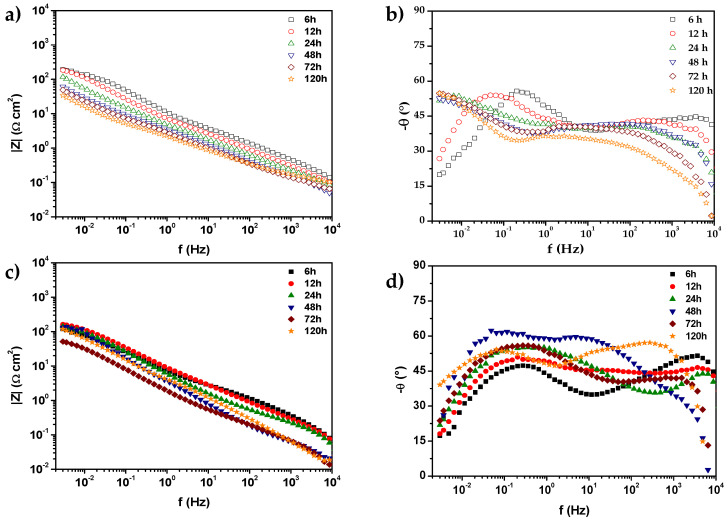
Corrected Bode plots of (**a**,**b**) 0% CeO_2_-NPs, and (**c**,**d**) 1% CeO_2_-NPs in Li_2_CO_3_-K_2_CO_3_ at 550 °C under an H_2_/N_2_ atmosphere.

Figure 7 shows the Bode diagrams of 0% CeO_2_-NPs and 1% CeO_2_-NPs samples after 120 h of exposure to Li_2_CO_3_-K_2_CO_3_ in an atmosphere of H_2_ at E = E_OC_ and 550 °C. The modulus impedance diagrams did not reveal significant differences in the LF range when CeO_2_-NPs were added. Note that in the sample with nanoparticles, the low-frequency impedance module decreases to 56.5 Ω cm^2^ after 72 h of exposure and then increases to 120.7 Ω cm^2^ after 120 h of exposure. This is likely associated with the oxide film cracks and then becomes passivated after 72 h. Figure 7b,d also show the phase angles for both samples. These do not present significant differences but reveal a capacitive response with two- or three-time constants in all frequency ranges, possibly associated with forming an oxide film [52].

Angles close to 20° are revealed at short exposure times at LF, 50° at longer exposure times at LF, and angles between 35 and 50° at HF. Due to the geometry of the electrode and surface heterogeneity, this response may be attributed to frequency dispersion [50]. Lee [53] studied the influence of temperature on the anode reaction in a molten carbonate fuel cell. Their results revealed that the high-frequency (HF) circle shrinks as the temperature increases, but the low-frequency (LF) circle increases. According to Lee [54], HFC represents cathodic mass transfer resistance through the electrolyte film, whereas LF represents gas-phase mass transfer resistance, mainly due to anode gas flow. Since the anode flow has a lower Reynolds number than the cathode, the anode has a much thicker boundary layer. The thickness of the layer causes mass transfer resistance in the gas phase. Therefore, the reduced size of HF indicates that the cathodic resistance decreases with increasing temperature. This is acceptable because an increase in temperature increases the solubility and diffusivity of the gas in the electrolyte film, thereby reducing mass transfer resistance.

**Figure 8:**
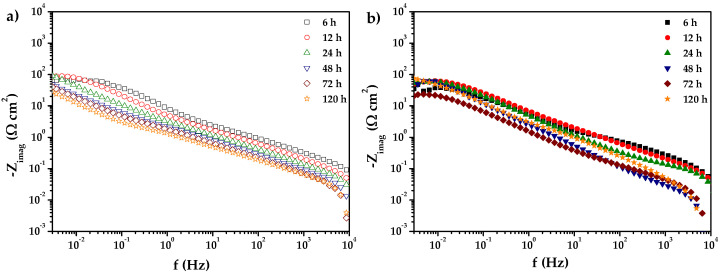
Log Z_imag_ of (**a**) 0% CeO_2_-NPs and (**b**) 1% CeO_2_–NPs in Li_2_CO_3_-K_2_CO_3_ at 550 °C under an H_2_/N_2_ atmosphere.

Figure 8 shows the variation of the imaginary part of the impedance of the samples with and without nanoparticles as a function of frequency after 120 h of exposure to Li_2_CO_3_-K_2_CO_3_ in a H_2_/N_2_ atmosphere at E = E_OC_ and 550 °C, revealing a constant phase element (CPE) behavior in the MF range, with a negative slope (α) varying between −0.45 ± 0.003 for 0% CeO_2_-NPs and −0.40 ± 0.04 for 1% CeO_2_-NPs, which can be related to the oxide film formed on the alloy surface (Z_oxide_) and described by the following relationship, as reported by Orazem and Tribollet [55], Tribollet et al. [56], and Hirschorm et al. [57].

(3) $$Z_{oxide}=\int_{0}^{\delta }\frac{\rho (\gamma )}{1+j\omega \rho (\gamma )\varepsilon (\gamma )\varepsilon_{0}}\;d\gamma$$

The authors proposed the power law model (PLM) to analyze the physical properties of the film as described by the Equation (4):

(4) $$Z(\omega )=g\frac{\delta \rho_{\delta }^{1-a}}{(\rho_{0}^{-1\;}{+j\omega \varepsilon \epsilon_{0})}^{a}}$$

In this case, α is the slope in the Log Z_Imag_ vs Log f graphs, ε represents the dielectric constant of the oxide layer formed on the metal alloy, ε_0_ is the vacuum permittivity, which has a value of 8.85 × 10^−14^ F‧cm^−1^, and g is a numerical coefficient close to 1 when α is 1, which can be estimated using the following equation:

(5) $$g=1+2.88\;(1-{a)}^{2.375}$$

Furthermore, *ρ*_0_ and *ρ_δ_* represent the lower and upper limits where the CPE behavior is observed in the frequency range. It should be noted that the Q value also corresponds to a parameter of the CPE, which relates to the dielectric constant, resistivity, and film thickness. The following equation can determine it:

(6) $$Q=\frac{{\left(\varepsilon \varepsilon_{0}\right)}^{a}}{g\delta \rho_{\delta }^{1-a}}$$

The graphical method for analyzing impedance data allowed us to estimate the Constant Phase Element (CPE) parameters and electrolytic resistance (R_e_). For the 0% CeO_2_-NPs sample, the Q coefficient was determined to be 6.87 × 10^−7^ F·cm^−2^‧s^−(1−α)^, with |α| corresponding to 0.44. In contrast, for 1% CeO_2_-NPs sample, the Q coefficient was found to be 3.62 × 10^−7^ F·cm^−2^‧s^−(1−α)^ with an |α| value of 0.50. The electrolytic resistance (R_e_) was assessed using a graphical method, resulting in approximately 0.935 Ω cm^2^ values for the 0% CeO_2_-NPs sample and 2.73 Ω cm^2^ for the 1% CeO_2_-NPs sample. These results demonstrate that adding nanoparticles results in an anodic behavior, accompanied by a re-passivation process at extended exposure times.

**Figure 9:**
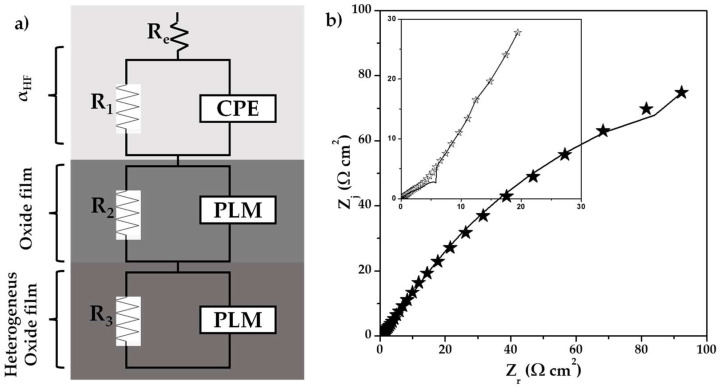
(**a**) A representative equivalent circuit of Cu-50Ni-5Al, (**b**) a comparison of ((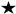) 0% CeO_2_–NPs, (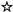) 1% CeO_2_–NPs) experimental, and (—) fitting adjusting of impedance data after 120 h of exposure in Li2CO_3_-K_2_CO_3_ at 550 °C under an H_2_/N_2_ atmosphere.

Figure 9a shows an equivalent circuit proposed for 0% CeO_2_-NPs and 1% CeO_2_-NPs after exposure to Li_2_CO_3_-K_2_CO_3_ at 550 °C under an H_2_/N_2_ atmosphere. Additionally, Figure 9b) shows the fitting results obtained by adjusting the experimental data, with χ^2^ = 1.8. It should be noted that the accuracy of the fitting is comparable to that of the graphic method.

### 3.3. Morphological and Microstructural Analysis

#### 3.3.1. Scanning Electron Microscopy Analysis

**Figure 10:**
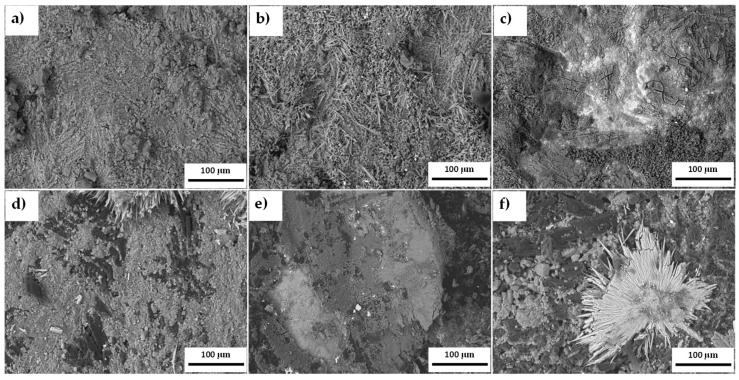
SEM images of the alloys (**a**–**c**) 0% CeO_2_-NPs, and (**d**–**f**) 1% CeO_2_-NPs.

The microstructure of the samples exposed to molten carbonate (Li_2_CO_3_-K_2_CO_3_ 62–38 mol.%) in an H_2_/N_2_ atmosphere is presented in Figure 10. Both alloys exhibit a mixed microstructure characterized by filament-shaped particles and a heterogeneous base corresponding to the surface’s aluminum and copper oxides. A significant surface modification was observed in the samples containing CeO_2_-NPs, leading to an increased formation of cracks and pores and a heterogeneous surface associated with the deposition of molten carbonates in the corrosion products. Additionally, the 1% CeO_2_-NPs sample exhibits a low presence of pores in the perforated area of the sample (see Figure 10f). Bright areas resembling needle-like structures corresponding to the microstructure of the molten carbonates were also observed.

**Figure 11:**
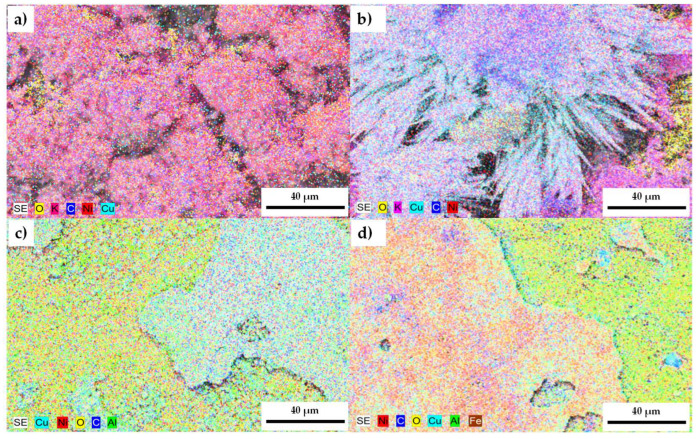
Mapping images of 0% CeO_2_-NPs and 1% CeO_2_-NPs. (**a**,**b**) After exposure to Li_2_CO_3_-K_2_CO_3_ at 550 °C under an H_2_ atmosphere and (**c**,**d**) with surface washing.

**Figure 12:**
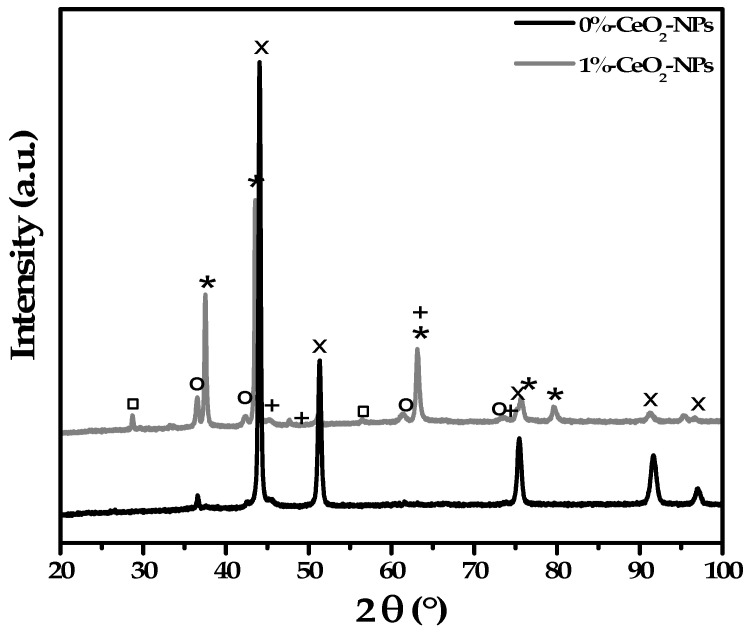
XRD patterns of 0% CeO_2_-NPs and 1% CeO_2_–NPs, after exposure to Li_2_CO_3_–K_2_CO_3_ at 550 °C under an H_2_/N_2_ atmosphere. (x) CuNiAl-SS; (□) CeO_2_; (*) NiO; (o) Cu_2_O; (+) Al_2_O_3_.

SEM/EDS mapping, as shown in Figure 11, provided information on the elemental distribution within both alloys, revealing areas with different shades. After 120 h of exposure, molten carbonates prevail on the surface of both alloys, concentrating oxygen and potassium in their vicinity (Figure 11a,b). Based on these results, the surface of the test pieces was cleaned to remove carbonates and to observe the heterogeneity of the elements that compose the alloy. Figure 11c,d show a clear segregation of the different elements resulting from forming their corrosion products (presented in XRD; see Figure 12).

At the grain boundaries, a higher concentration of copper is observed (Figure 11d), probably due to the natural tendency of copper–nickel alloys to undergo phase separation, indicated by the miscibility gap in their binary phase diagram [58]. This is accentuated in the samples by incorporating CeO_2_-NPs, where a Ni-rich zone, rich in Al and Cu, is observed. Furthermore, the formation of copper and nickel oxides in these regions may also contribute to the observed differences. This agrees with the results of EIS.

#### 3.3.2. X-Ray Diffraction Analysis

Figure 12 presents the diffraction patterns of the 0% CeO_2_-NPs and 1% CeO_2_-NPs after 120 h of exposure to molten carbonate. In the 0% CeO_2_-NPs sample, the diffraction pattern reveals a Cu-Ni-Al solid solution identified as typical fcc structures (Fm-3m) and minor phases of Cu_2_O (Pn-3m; JCPDS 010751531), and Al_2_O_3_ (R-3c; JCPDS 010772135). However, in the 1% CeO_2_-NPs NPs sample, the pattern shows the Cu-Ni-Al solid solution, the intriguing addition of CeO_2_ (Fm-3m; JCPDS 010750076), and the Cu_2_O and Al_2_O_3_ phases. Notably, NiO (Fm-3m; JCPDS 010731519) is also present, a surprising element absent in the absence of NPs. This is likely due to improved electronic coupling and interatomic interactions between CeO_2_ and NiO, which promote the kinetics of the process and electrochemical degradation [59].

#### 3.3.3. X-Ray Photoelectron Spectroscopy Analysis

**Figure 13:**
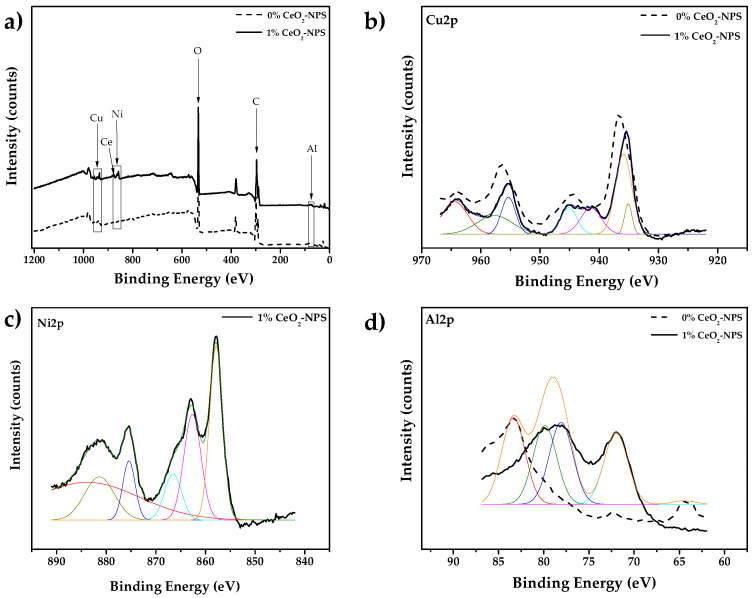
X-ray photoelectron spectroscopy (XPS) analysis (**a**) full scan spectrum of 0% CeO_2_-NPs and 1% CeO_2_–NPs, (**b**) Cu 2p, (**c**) Ni 2p, and (**d**) Al 2p.

Figure 13 presents the complete XPS spectrum of the 0% CeO_2_-NPs and 1% CeO_2_-NPs. In the case of the 0% CeO_2_-NPs sample, the deconvolution of the peaks reveals signals related to Cu(OH)_2_ in the Cu 2p spectrum at binding energies of 935.9 eV, 940.9 eV, 942.3 eV, 943.8 eV, and 944.9 eV. Cu(OH)_2_ possesses numerous electrochemically active sites, excellent energy storage capabilities, and a high surface area. Recent studies have shown its potential applications as a bacterial photocatalyst and in the electrochemical storage of hydrogen [60]. The Al 2p signal is observed as Al metal (at binding energies of 72 eV and 74.2 eV) and overlaps with Al_2_O_3_ (75.89 eV), indicating the presence of a passive layer on the surface of the alloy [61]. Notably, the Ni 2p signal was not detected in the XPS analysis. In corrosive environments, copper oxides (CuO, Cu_2_O) form preferentially on the surface of Cu-Ni alloys, while nickel can be incorporated into the copper oxide structure or remain on the substrate, making it difficult to detect by XPS [62]. Therefore, the primary corrosion products in this alloy consist of copper oxides (both I and II) and aluminum oxide.

The deconvolution of the peaks for the 1% CeO_2_-NPs sample shows the presence of CuO at binding energies of 940.9 eV, 942.4 eV, and 944 eV, as well as Cu_2_O at 935.4 eV and 956.0 eV. This suggests that a passive layer is likely to form on the surface. The Al 2p signal corresponds to Al_2_O_3_, exhibiting peaks at 75.0 eV and 89.0 eV with low intensity, likely due to the small amount of aluminum present in the alloy. The Ni 2p signal is identified as nickel hydroxide (Ni(OH)_2_) in the presence of CeO_2_-NPs with binding energies of 857.8 eV, 863.0 eV, and 864.7 eV, and the twin peaks of Ce^3+^ and Ce^4+^ can be observed at 882.6 eV and 885.5 eV, respectively [63], as shown in Figure 13c. The variable oxidation states of cerium (Ce) contribute to an increase in oxygen vacancies. The presence of Ce^3+^ ions facilitates interactions between ceria and surrounding nickel, thanks to the lone electrons in the 4f orbital. Oxygen-rich cerium oxide can also donate lattice oxygen to nickel, helping maintain the nickel ion in a higher valence of Ni^2+^ in NiCeO_x_. This process enhances the catalytic activity of the proposed material as anode [64,65,66]. According to Wang et al. [64], the presence of oxygen vacancy clusters in CeO_2_ nanocubes significantly enhances its catalytic activity, which is attributed to improved oxygen mobility and surface reactivity. These oxygen vacancies serve as active sites that facilitate the adsorption and activation of oxygen species, which in turn promote oxidative reactions on the catalyst surface. Moreover, Kumar et al. [65] demonstrated that the concentration of Ce^3+^ states and surface oxygen vacancies strongly influences the electrochemical performance of CeO_2_ nanoparticles. Their work emphasizes that Ce^3+^ not only enhances the electrical conductivity of the material but also increases the reactivity of oxygen species at the surface. These activated oxygen species are crucial for the oxidation of Ni species, facilitating the stabilization and formation of NiO. In alignment with these findings, Xiao et al. [66] explored the effect of Pr doping in CeO_2_ to engineer oxygen vacancies and improve the dispersion of nickel species. They observed that the presence of Pr further increased the density of oxygen vacancies, enhancing the stability and catalytic efficiency of the CeO_2_-NiO composite. This supports the hypothesis that CeO_2_ actively contributes to the formation and stabilization of NiO through its redox properties and oxygen mobility. These findings align with several studies indicating that NiO, NiO(OH), and Ni(OH)_2_ are suitable materials for use as electrodes in fuel cells and batteries due to their catalytic properties [67,68,69], which confirms the results observed in the SEM images.

## 4. Conclusions

Mechanical alloying and spark plasma sintering (SPS) were employed to produce Cu–50Ni–5Al alloys reinforced with and without 1% CeO_2_ nanoparticles (NPs).

The progressive loading scratch tests demonstrate that adding 1 wt.% nanoparticles can significantly reduce the coefficient of friction by up to 50%. Furthermore, the presence of nanoparticles leads to shallower scratches, indicating an improvement in wear resistance. The findings further indicate that key material characteristics—such as porosity, hardness, and nanoparticle incorporation—significantly shape the material’s response to single scratch events.

Electrochemical impedance spectroscopy (EIS) assessed their electrochemical behavior on a molten carbonate (Li_2_CO_3_-K_2_CO_3_) in a hydrogen atmosphere at 550 °C for 120 h. The results indicated that incorporating CeO_2_ nanoparticles reduces polarization resistance but promotes the re-passivation of the material at a longer exposure time.

The formed corrosion products were analyzed using SEM-EDS, X-ray diffraction and X-ray photoelectron spectroscopy. In both systems, the corrosion products consisted of aluminum oxide and copper oxide. Furthermore, incorporating nanoparticles in an H_2_/N_2_ atmosphere decisively promotes the formation of nickel oxides as the predominant phase.

Therefore, incorporating CeO_2_ nanoparticles into the Cu-50Ni-5Al system increased wear resistance. Additionally, it produces an anodic behavior and a re-passivation process in a hydrogen atmosphere at long exposure times.

Understanding this impact is crucial for developing strategies to enhance the material’s durability in challenging environments such as MCFC.

## Figures and Tables

**Table 1:** Parameters of the dimensions and areas from the wear profiles for the three selected loads.

Sample	Load (N)	Depth (μm)	Width (μm)	A_1_+A_2_ (μm^2^)	A_3_ (μm^2^)	Pile-Up Ratio
0% CeO_2_-NPs	7	0.7 ± 0.10	31.6	9.7	13.9	0.70
	23	4.9 ± 0.06	84.3	111.5	277.0	0.42
	33	10.5 ± 0.02	124.7	45.3	842.0	0.05
1% CeO_2_-NPs	7	1.5 ± 0.12	50.8	7.5	50.9	0.15
	23	5.0 ± 0.04	87.7	53.23	280.3	0.18
	33	8.9 ± 0.03	116.2	49.5	673.8	0.07

## Data Availability

The data presented in this study are available on request from the corresponding author.

## Abbreviations

The following abbreviations are used in this manuscript:

SPS	Spark plasma sintering
EIS	Electrochemical impedance spectroscopy
NP	Nanoparticles
SEM	Scanning electron microscopy
XPS	X-ray photoelectron spectroscopy
XRD	X-ray diffraction
MCFCs	Molten carbonate fuel cells
SOFCs	Solid oxide fuel cells
Cu	Copper
Al	Aluminum
Ni	Nickel
CeO_2_	Cerium oxide
ICR	Interface contact resistance
BPR	Ball-to-powder ratio
F_t_	Tangential force
F_n_	Normal force
COF	Coefficient of friction
E_OC_	Open circuit potential
HF	High frequency
LF	Low frequency
C_dl_	Capacitance of the electric double-layer
CPE	Constant phase element
PLM	Power low model
R_e_	Electrolytic resistance
